# Calorie Estimation From Pictures of Food: Crowdsourcing Study

**DOI:** 10.2196/ijmr.9359

**Published:** 2018-11-05

**Authors:** Jun Zhou, Dane Bell, Sabrina Nusrat, Melanie Hingle, Mihai Surdeanu, Stephen Kobourov

**Affiliations:** 1 Department of Computer Science Columbia University New York, NY United States; 2 Department of Linguistics University of Arizona Tucson, AZ United States; 3 Department of Computer Science University of Arizona Tucson, AZ United States; 4 Department of Nutritional Sciences University of Arizona Tucson, AZ United States

**Keywords:** calorie estimation, image annotation, crowdsourcing, obesity, public health

## Abstract

**Background:**

Software designed to accurately estimate food calories from still images could help users and health professionals identify dietary patterns and food choices associated with health and health risks more effectively. However, calorie estimation from images is difficult, and no publicly available software can do so accurately while minimizing the burden associated with data collection and analysis.

**Objective:**

The aim of this study was to determine the accuracy of crowdsourced annotations of calorie content in food images and to identify and quantify sources of bias and noise as a function of respondent characteristics and food qualities (eg, energy density).

**Methods:**

We invited adult social media users to provide calorie estimates for 20 food images (for which ground truth calorie data were known) using a custom-built webpage that administers an online quiz. The images were selected to provide a range of food types and energy density. Participants optionally provided age range, gender, and their height and weight. In addition, 5 nutrition experts provided annotations for the same data to form a basis of comparison. We examined estimated accuracy on the basis of expertise, demographic data, and food qualities using linear mixed-effects models with participant and image index as random variables. We also analyzed the advantage of aggregating nonexpert estimates.

**Results:**

A total of 2028 respondents agreed to participate in the study (males: 770/2028, 37.97%, mean body mass index: 27.5 kg/m^2^). Average accuracy was 5 out of 20 correct guesses, where “correct” was defined as a number within 20% of the ground truth. Even a small crowd of 10 individuals achieved an accuracy of 7, exceeding the average individual and expert annotator’s accuracy of 5. Women were more accurate than men (*P*<.001), and younger people were more accurate than older people (*P*<.001). The calorie content of energy-dense foods was overestimated (*P*=.02). Participants performed worse when images contained reference objects, such as credit cards, for scale (*P*=.01).

**Conclusions:**

Our findings provide new information about how calories are estimated from food images, which can inform the design of related software and analyses.

## Introduction

### Background

Estimating calories in pictures of food is an important task, providing data to inform nutrition research and practice and helping individuals achieve optimal, balanced dietary intakes. Yet this task turns out to be difficult for both experts and nonexperts. We are using this study as an opportunity to enhance our understanding of whether and how calorie estimation works “in the wild,” that is, in real-world scenarios. There are many applications of this understanding, ranging from improving the methodological rigor (and reducing the associated burden) of dietary assessment, a pervasive and unanswered question in nutrition science, to influencing the design of interventions focused on dietary behavior change.

The fact that individuals do not estimate calories well [1-4] has motivated the design of software apps to help individuals better estimate different aspects of dietary intake (eg, calories, energy density, nutrient density, and portions) using machine learning (ML) and by harnessing the “wisdom of the crowd.” The latter phenomenon was first documented in a 1907 Nature paper [5] and has been successfully used in many domains, ranging from gene network inference [6] to computational problems [7]. Apps in this space remain quite difficult to use, requiring burdensome manual logging of what one eats, or, when ML is used to classify pictures of foods, explicit weight values to be entered manually. To a large extent, the identification of calorie content from images of food either through crowd sourcing or ML remains an open research question. This work is a necessary step toward the automated identification of calorie content from images of food.

### Objectives

The aim of this study was to determine the accuracy of crowdsourced annotations of calorie content in food images and to identify and quantify sources of bias and noise as a function of respondent characteristics and food qualities (eg, energy density).

## Methods

### Procedure

The proposed task is essentially a combination of 2 tests individuals must engage in when estimating calories. The first test relates to the relative energy density of the food pictured, whereas the second test discerns the portion size. Thus, we contend that the ecological validity of our approach is high, despite the task’s complexity. The study protocol described herein was reviewed by an institutional review board at the University of Arizona and met the criteria for exemption under 45 CFR 46.101(b).

We designed a simple online quiz administered by a custom-built webpage to measure the accuracy of calorie estimation in pictures of food, verify the existence of collective wisdom, and analyze data and find patterns and trends that can be useful in the design of calorie-tracking apps.

We posted the quiz to SampleSize [8], a subreddit (ie, a forum on reddit) dedicated to posting surveys and survey results. This choice was made on the basis of having a large, active user base that reflects the demographics likely to make large-scale food annotations for reasons of personal interest in self-quantification.

The quiz began with a short introduction: “We would like to see whether you have a good understanding about calories. We will show you several pictures of food and your task, should you choose to accept it, is to guess how many calories are in the food. We will not share any identifying information about you. All of the data is anonymous.”

**Figure 1:**
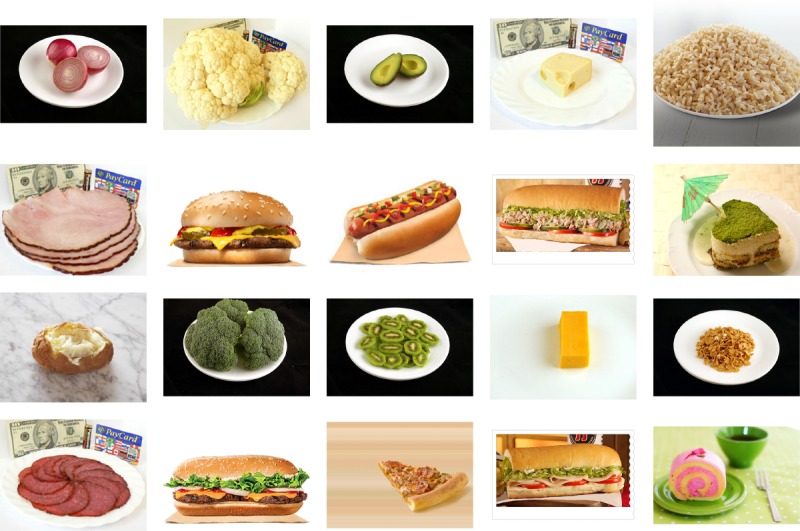
Untrained participants estimated the food calories in these 20 images.

The quiz included 20 questions. Each question consisted of a picture of some food item (see Figure 1) and the prompt, “How many calories are in the food pictured here? (Type a number in the box between 50 and 800).” Implausible dietary data, from (un)intentional under-reporting or over-reporting, are a pervasive problem in nutrition research and can introduce bias or lead to erroneous interpretations of diet-weight or diet-disease relationships. A common way of handling this issue is to exclude extreme values after the fact based on the distribution of the data (eg, removing data more than 2 SDs from the mean) or by subjective assessment [9]. In contrast, we provided the upper and lower limits on the guesses, based on the ground truth data, to ease an already difficult task and thereby reduce the amount of data that would later be necessary to remove. The numbers also helped clarify that we were referring to kilocalories and helped reduce outliers. Neither the correct calorie amounts nor other participants’ answers were visible to a participant during the estimation portion of the experiment, although it is possible that some might have read the reddit comments before participation, which revealed some calorie values. We decided to not add additional information to the pictures (eg, *does the sandwich contain mayonnaise?*) to keep the task closer to a realistic image annotation task.

Following the food-related questions, the participants were asked to provide their age group, gender, and body mass index (BMI). An option to calculate BMI via height and weight information was also available. We deliberately chose not to ask for additional demographic questions (eg, location, income, and education) to protect participant privacy. We reported the accuracy of the individual participant who just completed the quiz, as well as the average accuracy of all prior participants, using a breakdown showing the performance of each question.

### Quiz Materials

We used 2 categories of food for the pictures: single-ingredient (eg, broccoli, cheese) and mixed-ingredients (eg, sandwich, pizza). There were 20 pictures of food items in total (Figure 1): 12 single-ingredient and 8 mixed. The food shown in the pictures ranged from 100 to 720 kcal. Importantly, we chose these food items according to the United States Department of Agriculture’s (USDA) MyPlate model [10] that captures the building blocks for a healthy diet, and which includes 5 types of food (vegetables, fruits, protein, dairy, and grain), as well as mixed foods containing these ingredients. Our selection aimed to follow this model, to include realistic foods that appear in daily consumption, and to be concise so participants engage with the quiz.

The food portions selected are summarized in Table 1. The images were ordered so that each food type was maximally separated from other instances of its type, and the order was the same for each participant. We collected nutrition information about some food items from official restaurant websites. Although the calorie content of the foods pictured was not directly measured, US federal statute requires the published calorie values of restaurant food items to be within 20% of actual calorie value [11].

We chose not to inform the participants of the sources of the images, to reduce the potential that they would search the Web for “ground truth” data, for example, by going to the actual Burger King’s website. Likewise, the participants were not explicitly told that some images were from fast food restaurants.

### Patterns and Analysis

A total of 3 measures were relevant to our analysis:

1. Error, *e*, is estimated kilocalories (*ĉ*) minus ground truth kilocalories (*c*), *e* = *ĉ* − *c*, and percent error, *η*, is error as a percentage of the ground truth kilocalories, *η* = *e* / *c*, both of which are positive in overestimation and negative in underestimation. Because of the variation in the ground truth kilocalories of the foods, the latter is a more reliable indicator of the scale of response bias.
2. Absolute error, |*e*|, measures accuracy irrespective of the direction of estimation bias (|*e*| = |*ĉ* − *c*|).
3. Discrete accuracy, *D*, is the number of estimates that were within 20% of the true calorie value (out of 20 estimates); discrete accuracy was the measure reported to quiz participants:

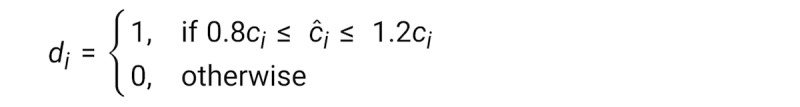

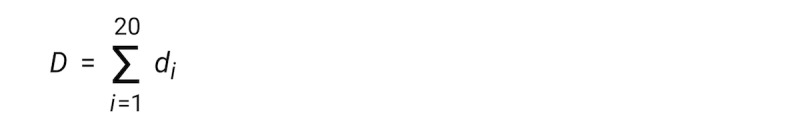

Before this analysis, we removed participants who reported a BMI less than 15 or more than 50 kg/m^2^ (which are unlikely to be correct), and participants who did not report their gender. In addition, we eliminated responses of less than 50 kcal or greater than 800 kcal and retained all the remaining ones.

We analyzed the results of the survey using linear mixed-effects modeling in R [21,22], allowing regression with random intercepts for both participants and foods simultaneously. The *R*^*2*^ values are the proportion of the variance in the data that is described by the models’ predicted values. For all analyses, a *P* value less than alpha=.05 was considered indicative of a statistically significant relation.

**Table 1:** Foods were chosen for the quiz to attain maximum coverage of food types encountered in daily life by likely participants. *Scaling* refers to the presence of reference objects, such as credit cards, which could indicate food volume.

Food	Type	Energy (kcal)	Mass (g)	Scaling?	Source
Cheddar cheese	Dairy	200	51	No	wiseGEEK [12]
Gouda cheese	Dairy	300	84	Yes	HealthAssist [13]
Avocado	Fruit	200	125	No	wiseGEEK [12]
Kiwi	Fruit	200	328	No	wiseGEEK [12]
Brown rice	Grain	420	297.7	No	Panda Express [14]
Cereal	Grain	200	55	No	wiseGEEK [12]
Ham	Meat	300	185.1	Yes	HealthAssist [13]
Salami	Meat	300	72.9	Yes	HealthAssist [13]
Red onion	Vegetable	200	475	No	wiseGEEK [12]
Potato	Vegetable	100	141.7	No	Food Network [15]
Broccoli	Vegetable	200	588	No	wiseGEEK [12]
Cauliflower	Vegetable	300	1200	Yes	HealthAssist [13]
Cheeseburger	Mixed	270	104	No	Burger King [16]
Hot dog	Mixed	310	123	No	Burger King [16]
Green tea cake	Mixed	136	40	No	Wit Co, Ltd [17]
Long cheeseburger	Mixed	590	213	No	Burger King [16]
Pepperoni and sausage pizza	Mixed	240	97	No	Papa John’s Pizza [18]
Swiss roll	Mixed	251	96	No	Slism [19]
Tuna sandwich	Mixed	720	420	Yes	Jimmy John’s [20]
Turkey sandwich	Mixed	510	254	Yes	Jimmy John’s [20]

## Results

In total, 2125 individuals participated in our reddit quiz. After removing 97 participants with missing or invalid demographic data, 2028 individuals were included in the analysis.

### Participant Demographics

**Figure 2:**
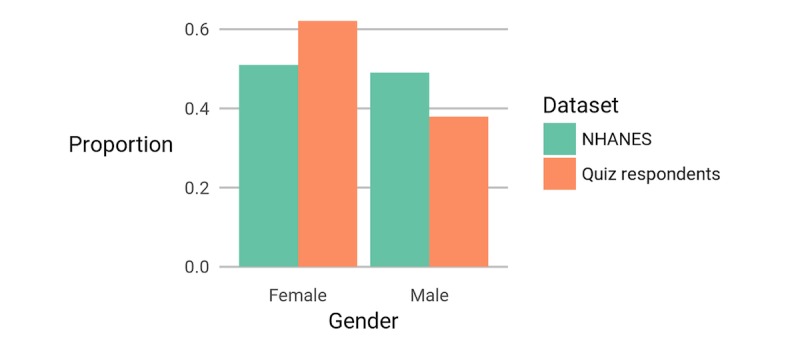
The reported gender of respondents to our quiz is compared with data from National Health and Nutrition Examination survey (NHANES).

**Figure 3:**
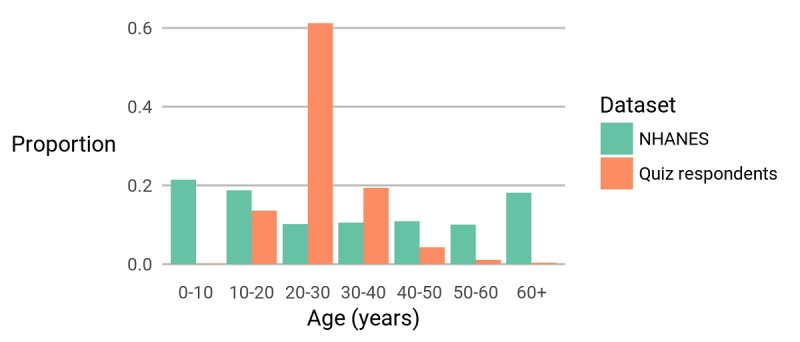
The age of respondents to our quiz is compared with data from National Health and Nutrition Examination survey (NHANES).

**Figure 4:**
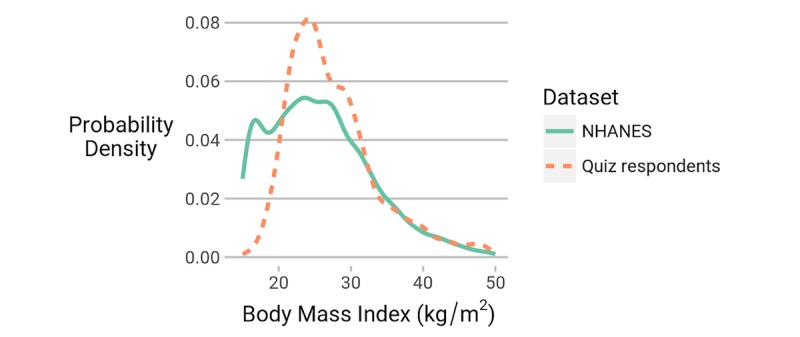
The body mass index (kg/m²) of respondents to our quiz is compared with data from National Health and Nutrition Examination survey (NHANES).

**Figure 5:**
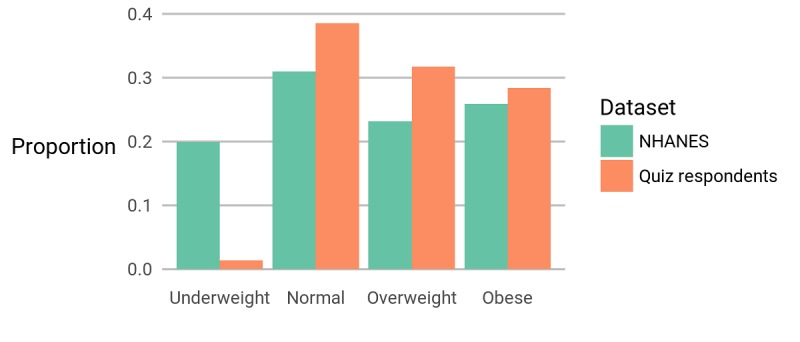
The body mass index (kg/m²) category of respondents to our quiz is compared with data from National Health and Nutrition Examination survey (NHANES).

The demographics of the participants are summarized in Figures 2-5. Although we collected no location data, an earlier study, again recruiting from the SampleSize subreddit, found that 67.4% (421/625) of participants reported a location within the United States [23], a rate that is similar to the 64% reported in another voluntary survey with participants from across reddit [24]. We also have a higher percentage of female participants than the US average, and a larger fraction of people with BMI around 25 kg/m^2^. It is possible that the participants in our quiz were more interested in this topic than the average person. However, in their self-selection, they are more demographically similar than the average person to likely crowdsourcing annotators for potential future app development.

### Participant Feedback

The participants volunteered their BMI and other demographic information, and 18 participants left 31 comments on the reddit thread. Table 2 summarizes the types of feedback comments we received, as well as some examples.

The feedback from the participants demonstrates engagement, interest, and curiosity. This implies that such tasks could be legitimately gamified (applying game mechanics and game design techniques to engage and motivate people to achieve their goals). It also shows that unlike Mechanical Turk participants, the participants in our study were engaged and motivated by intrinsic interest.

Note that our work addresses some of the requests shown in Table 2. For example, we found no increased accuracy from the presence of reference objects for scale in the pictures.

**Table 2:** Representative comments from the reddit post of the calorie estimation quiz.

Type	Example
Fun	“That was fun! I think the folks in ‘loseIt’ [another subreddit] and on various MFP [MyFitnessPal] forums would enjoy taking this, too.”
Surprise	“I’m really really doubtful that burger is only 270 cal.” “[N]o way are two red onions 200 calories.”
Units	“[C]ountries other than the US use the actual unit of energy- Joules”
Scale	“It would have been great to have a ruler next to the food.”
	“[I]f you show me a plate of rice, I can’t guess how much rice are on the plate because I don’t know how big the plate is.”
Difficulty	“Shoot, got 1 right out of 20 LOL. No wonder my BMI is 29.”
	“I dont know if there was mayo on [the submarine sandwiches] or not, which changes things a lot.”

#### How Good Are People at Estimating Calories?

**Figure 6:**
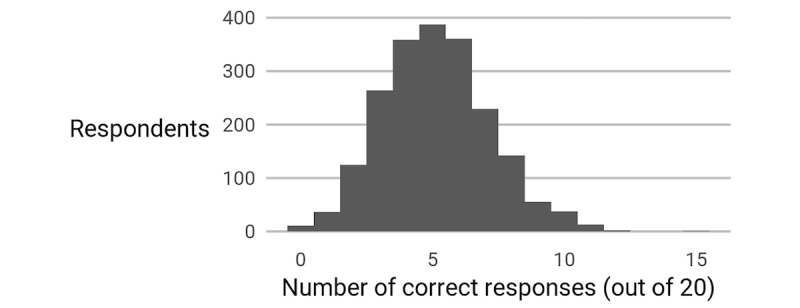
A histogram of the number of correct estimates each participant made. See Patterns and Analysis for the definition of this measure, D.

**Figure 7:**
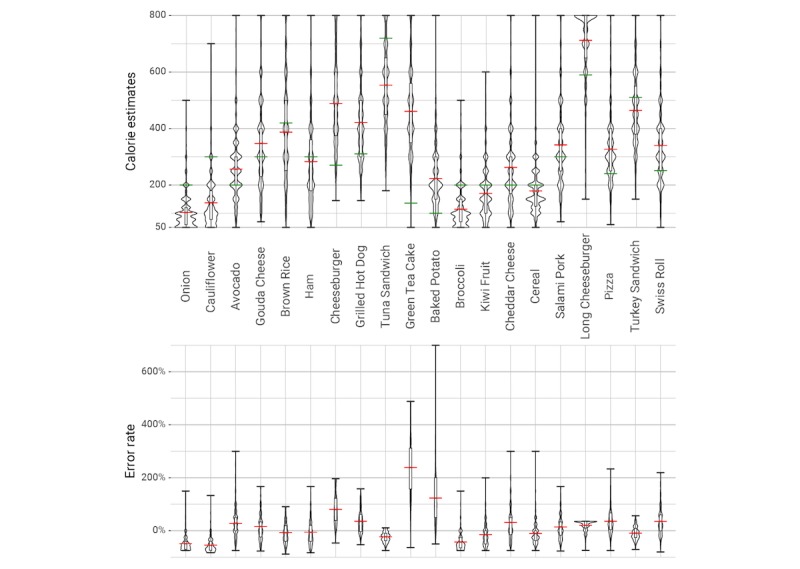
Calorie estimates and percent error (η) for each food item. For each food item, the violin plots represent the distribution of the calorie estimates by the participants and their percent error. The bottom and top of the boxes represent the first and third quartile, and the red band represents the mean of the calorie estimates, respectively. The green band represents the actual calorie value for each food item.

The participants' estimates had a mean absolute error (|*e*|) of 57.9% (136 kcal). In terms of discrete accuracy (*D*), the mean participant answered 5.15 questions correctly out of 20. Figure 6 shows the distribution of correct responses. Absolute error varied considerably by item from the most accurate item—a turkey sandwich, with a mean absolute error of 23.0% (39 kcal)—to the least—green tea cake, at 241.0% (327 kcal) absolute error. Figure 7 illustrates the variety of estimates and percent error (*η*) distributions for different items. Together, these facts show that human calorie estimates are both inaccurate overall and inconsistent in their inaccuracies.

#### Does the Wisdom of the Crowd Phenomenon Apply Here?

**Figure 8:**
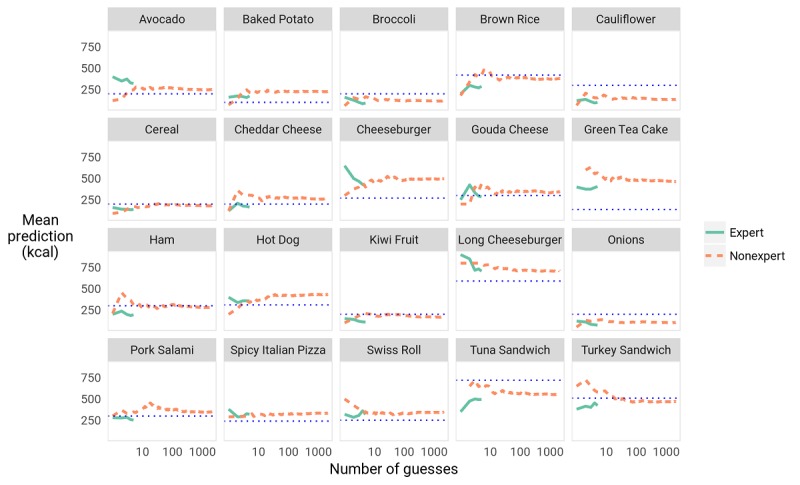
Mean estimates for each food as more participants are added show that a consensus forms rapidly. The dotted blue lines show the true calorie value for each food. The x-axis uses a logarithmic scale. The orange dashed line indicates the estimates of nonexperts. The green continuous line represents the estimates of nutrition science experts. Note that the range of acceptable calorie estimates was 50 to 800 calories for each food item.

A consensus formed rapidly for each food, as shown in Figure 8 (see the dashed orange line in the figure), so that 10 responses gave a very good estimate of the next 1000 responses. In fact, a bootstrap significance test shows that the average of 10 randomly selected participants’ guesses is no more (or less) accurate than the average of those of 1000 random participants (*P*=.36). Moreover, the consensus responses had greater discrete accuracy (*D*) than that of the individual participants, achieving 7 correct responses out of 20, a 36% relative improvement over the 5.15 correct among individual participants. This result is consistent with previous studies demonstrating the wisdom of the crowd, in which the accuracy of consensus judgments exceeds that of individual judgments (see Comparison With Prior Work).

Another important observation is that although error was high for individual responses and individual foods, the bias in the errors was low overall across all questions, such that the median of the error across items and participants is 0 (when using crowdsourcing over 2028 participants). Although this result is not actionable in itself, as it is averaged across all questions, it does demonstrate the power of crowds to converge toward high-accuracy judgments.

**Figure 9:**
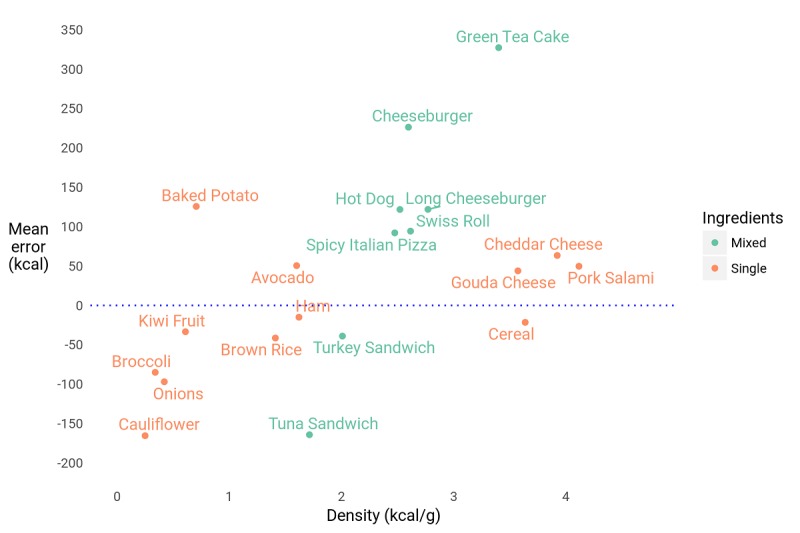
Participants underestimated the calorie content of calorie-sparse foods and overestimated that of calorie-rich foods.

#### Do the Nutritional Experts Outperform the Crowd?

In addition to redditors, we solicited participation from 5 nutritional experts. We recruited a faculty on a voluntary basis from the Department of Nutritional Science at the University of Arizona and the School of Nutrition and Health Promotion at Arizona State University. Somewhat surprisingly, neither the absolute error of their responses nor their discrete accuracy was statistically different from those of the average nonexpert participant (*P*=.19). In fact, a small crowd of only 2 randomly selected nonexperts was required to outperform the highest performing expert, achieving an average absolute error (|*e*|) of 119.3 (52.3%) compared with the expert’s 130.2 (55.3%). Expert performance is shown in comparison with nonexpert performance in Figure 8. This result is consistent with the hypothesis that the sources of error (eg, erroneous volume estimation due to a notion of typical portion size) apply equally to experts and nonexperts. Prior work in many domains of estimation has supported the notion that a relatively small group of nonexperts can estimate just as well as a single expert [25,26] (see also Comparison With Prior Work).

#### Does Having an Object for Scale in the Picture Help?

Several comments in the reddit thread expressed the hypothesis that pictures featuring a standard-sized reference object (such as a credit card) were easier to answer. The results showed that reference objects, far from aiding estimation, increased absolute error (|*e*|) by a mean 4.6 kcal (*P*=.01, *R*^*2*^=.31). Our hypothesis is that participants used background knowledge about the typical size of foods to scale foods but were not able to profit from comparison against the reference objects. This is statistically significant evidence for the notion that scale information does not aid calorie estimation in digital images (compare [27]). However, it is important to note that this was a post hoc analysis only; the experiment was not designed to analyze this hypothesis. For example, we included objects that come in many different sizes (eg, forks) as reference objects, which may have confused the quiz takers. We leave a more careful evaluation of this particular observation as future work.

#### Does Energy Density of Foods Predict Estimation Error?

As shown in Figure 9, the caloric content of energy-dense foods was systematically overestimated, and that of energy-sparse foods underestimated, as measured by error (*e*, *P*=.02, *R*^*2*^=.57). This bias is similar to one found by Almiron-Roig et al [28] in estimating in-person portion sizes and could reflect 2 nonexclusive sources. First, it could result from the perceived healthiness of the food items [29]. For example, broccoli is a prototypically healthy food but is not devoid of calories; conversely, prototypically unhealthy foods such as cheeseburgers have often been “engineered” for low calories [30]. This explanation aligns with the results of Carels et al [1], who found that college students overestimated the caloric content of foods considered to be unhealthy while they underestimated the number of calories in healthy foods. Second, the bias could result from an assumption that the items would have a similar weight to one another, when in fact there was an inverse relationship between the energy density and weight of the items (Pearson correlation: *ρ*=–.70). We hypothesize that inelastic adjustment of portion size according to energy density could contribute to obesity.

#### Does Body Mass Index Predict Estimation Errors?

BMI itself does not predict accuracy or bias in these data, similar to Blake et al [31] and Chandon and Wansink [32]. Other studies show that overweight and obese individuals consistently under-report calorie intake to a greater degree than nonoverweight individuals [33,34]. However, BMI does significantly interact with energy density in predicting percent error (*η*, *P*=.002, *R*^*2*^=.57), such that the higher a participant's BMI, the more they exaggerated the calorie content of calorie-rich foods. We hypothesize that overweight individuals are more sensitive to perceptions of food.

#### Do Gender and Age Predict Estimation Errors?

**Figure 10:**
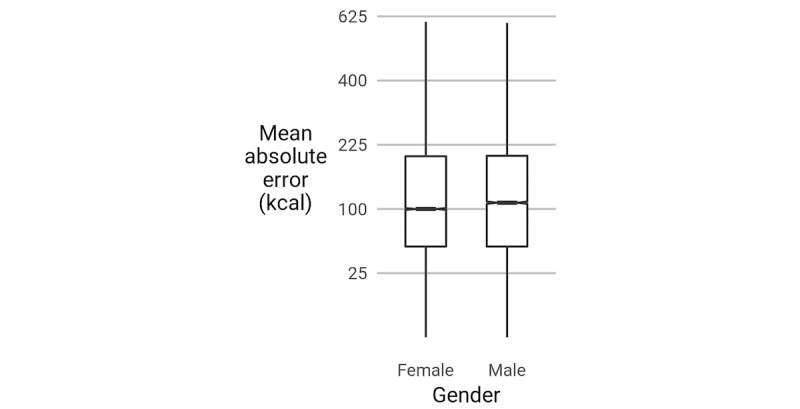
The absolute error (|e|) of participants differs by gender. Box edges show the first and third quartiles and are split by the median. The boxes’ whiskers extend to the farthest point within 1.5 times the interquartile range from the box ends. The notches denote the 95% CI of the median. The y-axis is on a square-root scale.

**Figure 11:**
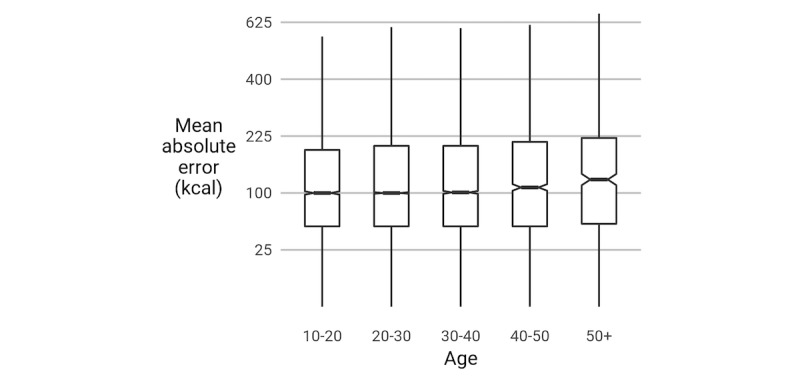
The absolute error (|e|) of participants differs by age. Box edges show the first and third quartiles and are split by the median. The boxes’ whiskers extend to the farthest point within 1.5 times the interquartile range from the box ends. The notches denote the 95% CI of the median. The y-axis is on a square-root scale.

No biasing effect (toward underestimation, for example) was found, but absolute error (|*e*|) was greater for men than for women (*P*<.001, *R*^*2*^=.31), similar to the portion judgment result by Almiron-Roig et al [28]. In addition, the absolute error was greater for older participants (*P*<.001, *R*^*2*^=.31), but these effects did not interact. Figures 10 and 11 summarize these differences. We hypothesize that the primary reason for these differences is cultural, reflecting gender norms and the relatively recent cultural emphasis on calories as a measure of healthiness.

#### Do Estimation Errors Cluster by Food Type?

**Figure 12:**
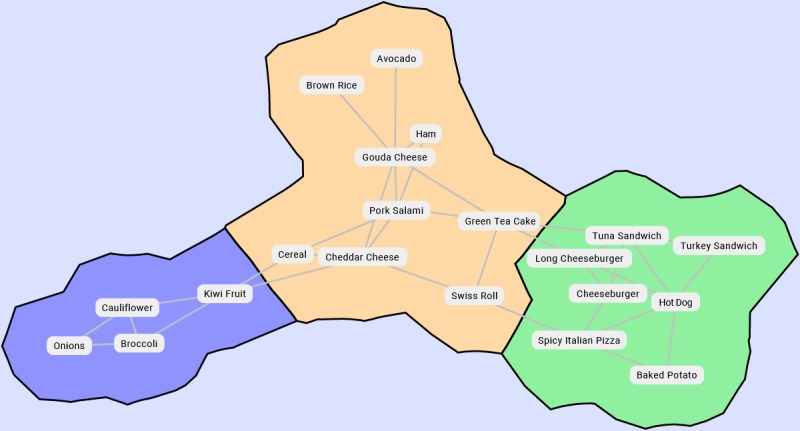
This map shows a network of food items in our survey based on correlation of estimation errors. Pairs of strongly correlated foods are connected by edges. The stronger the correlation, the closer the two (distances are inverse to correlation) are. Clusters show groups of similar subnetworks.

The over- and underestimation of errors for some foods correlate with those for others. For example, a participant who underestimates the calories in broccoli is likely to do so for cauliflower as well. Figure 12 shows an automatically generated map [35] illustrating these correlations, with clusters showing similar subnetworks. A larger map with more food items would be a strong basis for predicting human bias on clusters of food types (eg, vegetables).

## Discussion

### Principal Findings

The above analysis identifies several patterns that are important for the design of calorie estimation apps.

First and foremost, our study demonstrates that individuals are poor judges of calorie content in images, and prior work has shown that they are poor judges of portion size in real-life situations (see Comparison With Prior Work). This suggests the utility of an ML approach to calorie estimation to facilitate meal planning. Keeping track of calories by describing foods and guessing quantities and values is a tedious and inaccurate strategy, yet it is the strategy most commonly used in apps today. Given that “a picture is worth a thousand words,” our initial hypothesis was that using images (rather than descriptions of foods) should lead to better estimates. Our results, however, do not support this hypothesis: on average, participants performed poorly at estimating the amount of calories in pictures of food, answering 5.15 of 20 questions correctly on average. Our analysis indicates that participants in our dataset tended to exaggerate common dietary knowledge; they underestimated the number of calories in energy-sparse foods and overestimated them in energy-dense ones.

Our related work discussion (Comparison With Prior Work, below) highlights that estimating calories using ML remains an open research problem. However, our work suggests that such apps could take advantage of the wisdom of the crowd for estimation. We showed that the crowd performs better than experts, on average, even when the crowd is small. This suggests that this annotation could be implemented accurately and at low cost.

The results suggest that for apps that focus on calorie monitoring (including self-reporting), it might be a good idea to characterize the users’ demographic data (age, gender, and BMI) shown to influence the accuracy of calorie estimates either directly or when combined with other factors such as energy density.

We identified additional patterns that simplify the design and implementation of calorie-tracking apps. The first such pattern is that scale information does not improve estimation accuracy. The second is that estimation errors cluster by food types, which indicates that the app may extrapolate user patterns between foods in the same group.

It is important to note that the observations of this study are statistically significant and applicable to the population of interest to us (ie, individuals likely to participate in crowdsourced annotations). This population is considerably younger than the US population (χ^2^_6_=3362.5, *P*<.001) and contains more women proportionally (χ^2^_1_=81.0, *P*<.001). In future work, we aim to repeat this study for a larger population that matches known demographics to verify the validity of our analysis on such populations.

### Comparison With Prior Work

Related work includes prior work in nutritional sciences, ML, image processing, and crowdsourcing. We review a small but representative subset below.

#### Nutrition and Diet

Bandini et al [36] and Schoeller et al [37] have reported that individuals tend to selectively under-report the energy intake when these data are manually logged. This seems to be especially true for overweight and obese individuals [33,34] and could be associated with a failure to accurately estimate portions, although Blake et al [31] and Chandon and Wansink [32] found that BMI does not correlate with the ability to estimate calories when this task is conducted in person. Portion estimation of in-person food remains poor, whether in reference to images on computer screens or on printed images [38]. However, calorie estimation of large meals may be worse than that of small meals [39].

To monitor dietary intake more accurately, third-party automated food analysis systems have been proposed. Martin et al [40] used the remote food photography method (RFPM), which requires individuals to upload 3 pictures when having a meal: the plate of the foods selected by an individual, standard portions of known quantities of the foods, and the leftovers. These pictures are sent to trained dietitians who verify portions with participants and analyze these data using a standardized nutrient database. This approach relies on the judgment of trained nutrition professionals and argues for the validity of RFPM. Providing all 3 pictures for each meal is a challenge, as indicated by Williamson et al [41]. Beltran et al [42] tested the reliability of the eButton system, in which a camera worn on the chest records images continuously. The images are captured passively while the participant goes about their day, but such a system still requires experts to identify foods in the images and confirm them with participants. Similar to the RFPM employed by Martin et al [40], the eButton system requires valid pictures before and after each meal, camera placement at a certain angle, and proper lighting. Although promising, such systems are unlikely to scale to the millions of people who would like to accurately track their nutritional intake.

#### Machine Learning and Image Processing

Given the challenges of the systems described above, a system that can automatically measure calories in pictures of food would be in great demand. Image processing techniques can be used to recognize food in images, and ML can be used to estimate the calories in the food.

Menu-Match [43] uses a database of restaurants and Global Positioning System locations and attempts to guess what is in the picture, using image features such as color and scale-invariant feature transforms [44]. It has not been made available to the general public. Im2Calories [45] is built on the work of Menu-Match. A multi-label classifier is trained on a collection of images of food. The app locates the restaurant a user is dining in and, given an image from the user, the classifier (running on the user’s phone) guesses which foods are present in the meal. Looking up the nutritional facts provided by the restaurant, using the resulting estimates, yields good results. Note, however, that Im2Calories has not been made available to the general public or even for research purposes.

Bettadapura et al [46] show that food recognition using location data improves accuracy. Such systems, however, are inherently limited to the restaurants whose menus are in the database. These also assume that menus do not change often and that the volume of food is the same from plate to plate. In reality, most meals are eaten either outside of restaurants or in restaurants whose menus are not included in some dataset. The “in the wild” problem is more natural but also more difficult.

The Web app Foodlog [47,48] divides food images into 300 blocks each and extracts discrete cosine transform coefficients and color histogram from each block. Using these data, Foodlog classifies the food into 5 categories according to the USDA's My Pyramid system. Experimental results report 88% accuracy in the extraction of food and 73% accuracy in food balance estimation. The FoodCam system [49] segments the region of each food by GrabCut (an image segmentation approach based on iterative graph-cuts) [50], extracts image features of histogram of oriented gradients [51] and color patches with the Fisher Vector (an image representation obtained by pooling local image features) [52], and finally classifies it into 1 of 100 food categories using linear support vector machines.

With the exception of Im2Calories, the systems above achieve relatively good food recognition but without volume estimation. To estimate volume, Chae et al [53] minimize the false-segmented regions, smooth the segmentation boundaries of food, and reconstruct 3D primitive shapes from a single food image. He et al [54] estimate the weight of food given a single image using a shape template for regular-shaped foods and area-based weight estimation for irregularly shaped food. The Im2Calories system [45] estimates the distance of every pixel from the camera by using a convolutional neural net architecture, converts the depth map into a voxel representation, and estimates the volume of the food. Although such approaches are effective, there is no app for estimation of food volumes available to the general public.

#### Crowdsourcing

Crowdsourcing sometimes makes it possible to use multiple nonexpert judgments to approach the high quality of expert annotation [55]. Surowiecki [25] argues that in many instances, the average nonexpert estimates can even outperform a single expert. Watson has shown that the average of the individual judgments can be equal or superior to the judgment of the best individual within the group [26]. Moreover, the validity of judgments increases with more judges [56]. The strength of the wisdom of the crowd over ML is well understood and exploited in industry. For example, CardMunch (now a service of Evernote [57]) uses crowdsourcing with Amazon Mechanical Turk to convert pictures of business cards into digital contact information. Eloquent Labs [58] uses a mix of crowdsourcing with an artificial intelligence to implement a conversational assistant for customer service.

In the nutrition domain, Mamykina et al [59] show that crowdsourced ingredient annotations from food images are improved by expert annotation and by showing the annotators previous annotations of the images. The PlateMate [60] app leverages crowdsourcing to implement the first step in the RFPM. Rather than typing names of foods and estimating portions, users take photographs of their plates both at the beginning of the meal and at the end to accurately capture how much food was actually eaten. PlateMate uses annotations from nonexpert Amazon Mechanical Turk workers instead of expert dietitians to estimate the composition of foods in static images. PlateMate's results are as accurate as the experts. Similarly, the Im2Calories [45] project uses crowdsourcing to annotate all the food terms that apply to an image. Manually merging synonymous terms, they create the Food201-Multilabel dataset for training. Compared with the original Food101 classes, the new classes of Food201-Multilabel do better according to mean average precision, as they often correspond to side dishes or small food items.

In sum, despite the abundance of interest in this and related topics, including calorie-tracking apps with manual entry, there exists no publicly available app that will accurately estimate calories from a single image. Likewise, although there are many studies of human bias in tracking calories and lack of skill in estimating portion sizes, no previous work establishes the accuracy and biases of crowdsourcing for calorie estimation, or what demographic factors might correlate with accuracy.

Learning from our study, we envision a very simple app, where the only action required from the user is to take a picture of her or his food. The estimation logic, driven by the wisdom of the crowd and ML, would be transparent to the user, that is, it would be triggered automatically when the camera is used. The logic includes (1) detecting if the picture is a picture of food using image classification [61,62] and (2) routing the image for crowd annotations (similar to CardMunch, which routes the task of processing images of business cards to the crowd). We hope that this simplicity will yield wide adoption, which, in turn, will lead to measurable effects in dietary choices.

### Limitations

Participants were not directly informed that some images were of fast food and thus more likely to be subject to food engineering, for example, replacing sugar or using sweetness enhancers, or adding water or protein to enhance food properties and palatability [30,63]. The fact that this was not explicitly mentioned to the participants raises the possibility that participants might have considered these foods as “homemade,” which may have influenced perceived energy density and calories. However, since a majority of hamburgers are eaten at restaurants rather than homemade, judgments about engineered foods are as or more relevant than home-cooked foods for both naturalistic and app-related purposes

### Conclusions

We described a study measuring the ability of over 2000 individuals to estimate calories in 20 pictures of food chosen to capture the building blocks of a healthy diet [10]. We believe this study should be read as an analysis that drives the design of future food-related apps, with additional impacts on crowdsourcing strategies and the design of human-computer interfaces.

Our analysis confirms some earlier observations (eg, calorie estimation is a difficult task, even for the experts) and offers new insights:

1. Even a small crowd of 2 nonexperts achieves calorie estimation accuracy greater than that of the expert annotators. This suggests that semiautomated food labeling apps can be implemented at a low cost by harnessing the wisdom of the crowd, even when the crowd is small. Note that some prior approaches in this space, such as PlateMate [60], use crowdsourcing to provide calorie information to users. To the best of our knowledge, the crowdsourcing method has never been tested as a source of data for algorithmic calorie estimation before.
2. We found new type-of-food effects, with energy-dense foods (such as hamburgers) being consistently overestimated and energy-sparse foods (such as broccoli) consistently underestimated. Future crowdsourcing (or ML) projects aiming to annotate food for calorie content will benefit from correction using these biases.
3. We found the absence of some expected correlations. For example, the presence of reference objects for scale does not improve accuracy but rather slightly decreases accuracy, and the BMI is not correlated with accuracy. These observations impact the design of interfaces for annotation apps, as well as data collection protocols.

All in all, this work suggests that calorie-estimation apps are needed and can be built at low cost (eg, using small annotator groups, and without the overhead of including reference objects in images, or controlling for the BMI of users).

Several interesting research questions remain. First, given the low calorie estimation accuracy (5 out of 20) and some clear patterns (underestimating “healthy” foods and overestimating “unhealthy” foods), it is natural to ask whether simple training with feedback can help improve accuracy for nonexperts. If so, how much training is required, what gains in accuracy can be obtained, and how much further can the crowd boost the results? Second, can we factor in biases (eg, age, gender) to obtain better crowdsourced prediction? Third, can better (more consistent) reference objects lead to improvements in accuracy? Fourth, assuming the baseline accuracy for “simple” foods (eg, fruits, vegetables, and sandwiches) can be improved with some of the ideas above, can we hope to tackle more difficult challenges, such as amorphous foods (porridge, mashed potatoes) and liquids (soups, smoothies) in which ingredients and volume are less obvious? Lastly, but perhaps most importantly, we aim to apply the knowledge gained from this study beyond the understanding of how (or how well) people estimate calories to include assessment of diet quality, which has become a dietary construct of interest in the past 5 years [64]. This change has occurred because dietary patterns and dietary quality (eg, increased nutrient density, nutrient diversity, and nutrient adequacy) have been strongly associated with health and disease outcomes. This information provides potentially more meaningful metrics than the number of calories (which says nothing about the quality or “healthiness” of the food) when providing participants or patients with feedback.

We believe this study should be read as an analysis that informs the design of future food-related apps (in particular, apps that feature calorie estimation), with additional potential impacts on crowdsourcing strategies and the design of human-computer interfaces. Our future goal is to provide estimates about judging calories from images for the purpose of mass annotation (eg, in support of a calorie-estimation app), which, in turn, is part of a larger system that analyzes text, images, and videos to estimate risk of diet-sensitive diseases such as type 2 diabetes mellitus [23,65].

## Abbreviations

BMI: body mass index
ML: machine learning
RFPM: remote food photography method
USDA: United States Department of Agriculture
